# Comparison of RT-PCR, RT-nested PCRs, and real-time PCR for diagnosis of severe fever with thrombocytopenia syndrome: a prospective study

**DOI:** 10.1038/s41598-021-96066-4

**Published:** 2021-08-18

**Authors:** Sehrish Jalal, Seong Yeon Hwang, Choon-Mee Kim, Dong-Min Kim, Na Ra Yun, Jun-Won Seo, Da Young Kim, Sook In Jung, Uh Jin Kim, Seong Eun Kim, Hyun ah Kim, Eu Suk Kim, Jian Hur, Young Keun Kim, Hye Won Jeong, Jung Yeon Heo, Dong Sik Jung, Jieun Kim, Sun Hee Park, Yee Gyung Kwak, Sujin Lee, Seungjin Lim, Sun Hee Lee

**Affiliations:** 1 Department of Bio-Medical Science, College of Medicine, Chosun University, Gwangju, Republic of Korea; 2 Department of Internal Medicine, School of Medicine, Chosun University, 588 Seosuk-dong, Dong-gu, Gwangju, 61453 Republic of Korea; 3 Premedical Science, College of Medicine, Chosun University, Gwangju, Republic of Korea; 4 Department of Internal Medicine, Chonnam National University Medical School, Gwangju, Republic of Korea; 5 Division of Infectious Diseases, Keimyung University Dongsan Medical Center, Daegu, Republic of Korea; 6 Department of Internal Medicine, Seoul National University Bundang Hospital, Seongnam, Republic of Korea; 7 Department of Internal Medicine, Yeungnam University Medical Center, Daegu, Republic of Korea; 8 Department of Internal Medicine, College of Medicine, Yonsei University Wonju, Wonju, Republic of Korea; 9 Department of Internal Medicine, College of Medicine, Chungbuk National University, Cheongju, Republic of Korea; 10 Department of Infectious Diseases, School of Medicine, Ajou University, Suwon, Republic of Korea; 11 Department of Internal Medicine, College of Medicine, Dong-A University, Busan, Republic of Korea; 12 Department of Internal Medicine, College of Medicine, Hanyang University, Seoul, Republic of Korea; 13 Department of Internal Medicine, College of Medicine, The Catholic University of Korea, Daejeon, Republic of Korea; 14 Department of Internal Medicine, Inje University Ilsan Paik Hospital, Goyang, Republic of Korea; 15 Department of Internal Medicine, College of Medicine, Pusan National University, Yangsan, Republic of Korea; 16 Department of Internal Medicine, School of Medicine, Pusan National University, Pusan, Republic of Korea

**Keywords:** Biological techniques, Molecular biology

## Abstract

We designed a highly sensitive reverse transcription nested polymerase chain reaction targeting the M-segment (NPCR-M) of severe fever with thrombocytopenia syndrome (SFTS) virus. NPCR-M was performed in parallel with three other referenced PCR assays QPCR-S, PCR-M, and NPCR-S to assess their clinical usefulness as routine diagnostic techniques for SFTS. In this multi-centered prospective study, 122 blood samples from 38 laboratory-confirmed SFTS patients and 85 control samples were used. The results demonstrated that QPCR-S and NPCR-S had better sensitivity rate up to 21 days after symptom onset however, the PCR-M showed poor sensitivity after 7 days of symptom onset. Our designed NPCR-M had a higher detection rate up to 40 days from symptom onset and revealed the persistence of SFTSV RNA in the early convalescent phase. No false-positive results were seen for the control samples. Additionally, NPCR-M showed positive results for a sample that initially showed negative results from other PCRs and for many other samples collected in the convalescent phase of SFTS. Our designed nested PCR is suitable for SFTSV detection in patients’ blood collected in the acute and early convalescent phase of SFTS, and shows better sensitivity and high specificity even up to 40 days after symptom onset.

Severe fever with thrombocytopenia syndrome (SFTS) is an emerging viral disease caused by a novel bunyavirus, of the genus Phlebovirus in the family Bunyaviridae, endemic to China, Japan, and Korea^1–3^. SFTS virus (SFTSV) is transmitted to humans through tick bites, and *Haemaphysalis longicornis* is known to be a primary vector^4^, although human-to-human transmission has been reported^5^. The virus is an enveloped, negative-stranded RNA type, and the genome consists of three RNA segments designated L, M, and S. The L segment contains 6368 nucleotides encoding an RNA-dependent RNA polymerase, the M segment contains 3378 nucleotides encoding precursor glycoproteins N and C (GnGc), and the S segment contains 1744 nucleotides of ambisense RNA encoding a nonstructural protein (NSs) and a nucleocapsid protein (N) in opposite orientations^6, 7^. The clinical features of SFTS are characterized by nonspecific symptoms and signs, including the abrupt onset of high fever, gastrointestinal symptoms, severe malaise, thrombocytopenia, leukocytopenia, multi-organ dysfunction, and hemorrhagic tendency, in severe cases. Abnormal laboratory findings share several features with other viral hemorrhagic fevers and need to be differentiated from various infectious diseases, in particular, hemorrhagic fever with renal syndrome (HFRS) caused by hantavirus, human anaplasmosis, and dengue fever^1, 8^. Hence, laboratory confirmation is essential. Isolation of the virus in cell culture must be conducted within a bio-safety level 3 (BL3) facility. The virus may have little or no cytopathic effect; therefore, confirmation by electron microscopy and molecular or serological methods is needed. Specific antibodies to SFTSV are detectable approximately 7 days after disease symptom onset. Several serological methods are used to detect antibodies against viruses, including indirect immunofluorescence assay (IFA), ELISA, and the serum neutralization test (SNT). These techniques are time-consuming and require trained personnel and special equipment^9^. It can be difficult to obtain results in the acute phase when the antibody titer is below the detectable limit. Reverse transcription-polymerase chain reaction (RT-PCR) for laboratory diagnosis of SFTSV infection is a specific, sensitive method to detect viruses in the acute phase. A further advancement was the development of automated real-time assays, which provide higher sensitivity and specificity, show minimal carry-over contamination, and require less time than conventional RT-PCR^7, 10^.

SFTS is considered an increasingly important threat to regional as well as global health, having a high fatality rate^11^ and potential for person-to-person transmission. Therefore, reliable, rapid, sensitive, and specific laboratory diagnostic methods are essential to meet the needs of clinical SFTS case identification and for the prevention of spread in the community. However, there have been no studies comparing the reliability of these diagnostic PCR methods to detect SFTSV in the same specimen of clinically and laboratory-confirmed SFTS patients.

In the present study, we developed a useful reverse transcription nested polymerase chain reaction (RT-N-PCR) and compared it to three different polymerase chain reaction (PCR) assays for the detection of SFTS virus from patient samples to evaluate their potential use for rapid and accurate laboratory diagnosis of SFTS.

## Results

### SFTS patient laboratory test confirmation

A total of 259 patients fulfilled the criteria for possible SFTS infection and were enrolled in this study. Of these, 38 patients with a clinical presentation of SFTS were confirmed by positive PCR, seroconversion, and virus isolation. Seroconversion was recorded in 31 of the patients, and 13 patients were confirmed through successful SFTSV isolation. Among the 8/38 patients admitted to the hospital within 3 days of symptom onset, 21 were admitted between days 4–7, and the remaining 9 were admitted between days 8–14 of onset of symptoms. Initial samples were defined as samples collected on the first day of hospital admission. Additionally, 84 follow-up samples were collected from 32 SFTS confirmed patients, bringing the total to 122 samples analyzed in this study.

### PCR comparison

**Figure 1:**
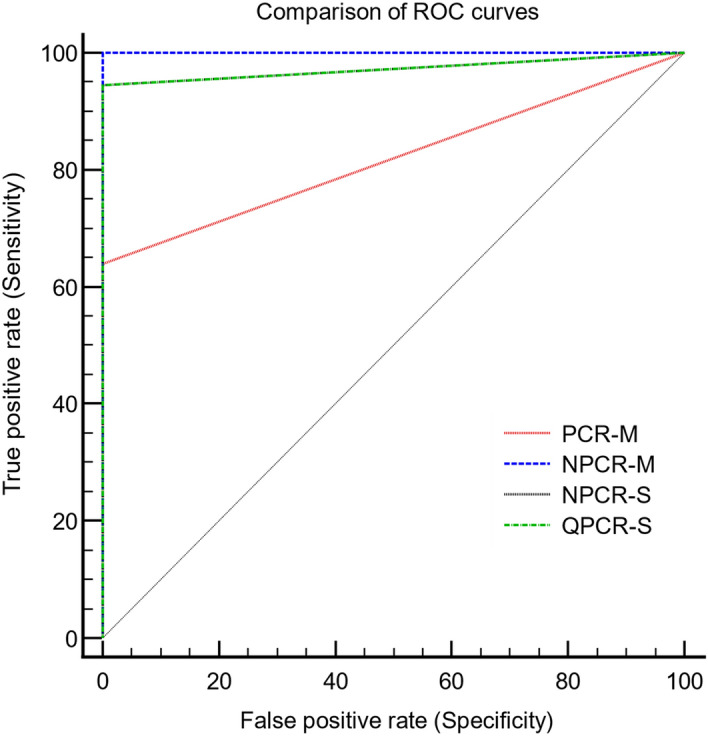
Comparison of receiver operating characteristic (ROC) curves of various PCRs analyzed in this study. Single-round PCR-M = reverse transcription (RT) polymerase chain reaction (PCR) targeting the M segment of the SFTS virus. NPCR-M = RT-nested PCR targeting the M-segment of the SFTS virus. NPCR-S = RT-nested PCR targeting the S-segment of the SFTS virus. QPCR-S = Real-time RT PCR targeting the S-segment of the SFTS virus.

**Figure 2:**
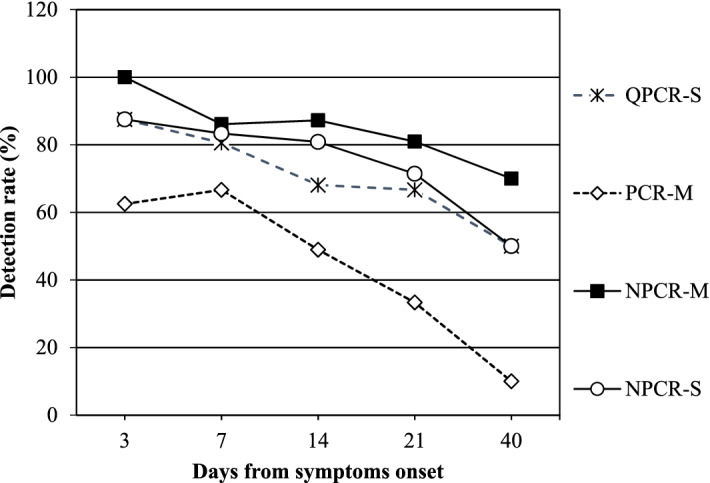
Time kinetic study of various PCR assays using severe fever with thrombocytopenia syndrome (SFTS) patient samples after symptom onset day. Single-round PCR-M = reverse transcription (RT) polymerase chain reaction (PCR) targeting the M segment of the SFTS virus. NPCR-M = RT-nested PCR targeting the M-segment of the SFTS virus. NPCR-S = RT-nested PCR targeting the S-segment of the SFTS virus. QPCR-S = Real-time RT-PCR targeting the S-segment of the SFTS virus.

A total of 122 clinical specimens from 38 SFTS laboratory confirmed patients were analyzed by the NPCR-M method, and the results were compared with those obtained by single-round PCR targeting the M-segment (single round PCR-M), QPCR-S, and NPCR-S. Using SFTS patients’ initial samples collected at the time of hospital admission (n = 38), single-round PCR-M yielded a positivity rate of 63% (24/38 samples) with a sensitivity of only 63.9% (95% confidence interval [CI], 46.2–79.2 ) specificity of 100% (95% CI, 95.7–100), and an area under the curve (AUC) of 0.819 (95% CI, 0.739–0.883) using MedCalc statistical analysis software for receiver operating characteristic (ROC) curve analysis. In contrast, second-round amplification by NPCR-M generated a positivity rate of 97.3% (37/38) with a sensitivity of 100% (95% CI, 90.3–100), a specificity of 100% (95% CI, 95.8–100), and an AUC of 1 (95% CI, 0.985–1.000). ROC curve comparison of these two PCRs showed differences between areas of 0.181(Standard error SE ± 0.0406) (95% CI, 0.101–0.260) with statistically significant *P* < 0.0001. None of the healthy or non-SFTS patients showed any positivity by either assay. Taking a negative cutoff value of 39 Cp/Ct for QPCR-S, and for the NPCR-S assay the positivity rate for both was 92.1% (35/38) with a sensitivity of 94.4% (95% CI, 81.3–99.3), both having specificity of 100% (95% CI, 95.8–100), and an AUC of 0.97 (95% CI, 0.925–0.994). The ROC curve comparison of both QPCR-S and NPCR-S with NPCR-M showed differences between areas of 0.0278 (± 0.0194) (95% CI, 0.101–0.260) with P = 0.1513. ROC curve comparison of both QPCR-S and NPCR-S with PCR-M showed differences between areas of 0.153 (± 0.0389) (95% CI, 0.0765–0.229) with significant *P* = 0.0001. (Table 1).The ROC curve comparison of the PCRs analyzed in this study is presented in Fig. 1. One of the patient’s initial samples collected on day 4 was negative for all the PCR assays except NPCR-M. Additionally, the follow-up samples were also negative for all assays. This sample was confirmed by sequencing, and notably, we confirmed the seroconversion for the convalescent-phase serum samples for that patient further supporting the result obtained by the NPCR-M assay. This suggests that NPCR-M has the potential for increased sensitivity, leading to earlier detection of infection. Considering the total of 122 samples, including 84 follow-up samples from 32 patients over a period of 40 days, single-round PCR-M showed very low detection ability with only 60 (44%) samples being positive, whereas NPCR-M showed that 104 (85%) samples were positive. QPCR-S detected 87 (71%) samples and NPCR-S detected 95 (75%) samples as being positive. Comparing the M-segment targeting PCR assays for SFTSV detection in blood samples in relation to days after symptom onset, the single round PCR-M consistently exhibited a lower SFTSV detection rate with statistical significance *P* = 0.02, whereas the NPCR-M demonstrated an enhanced detection rate throughout the 40 days of the study period and the results were not statistically significant (*P* = 0.44). This correlation suggests that N-PCR M sensitivity (detection rate) is independent of the day after symptom onset when the sample is collected. Hence, it is useful for SFTSV detection in samples collected in both the acute and early convalescent phases. Comparing the S-segment targeting PCR assays, QPCR-S and NPCR-S presented a comparable higher detection rate for samples collected between 1–7 days. While the NPCR-S maintained its high detection rate, a gradual decrease in the QPCR-S detection rate was observed for samples taken between 8–21 days, and then the detection rate markedly decreased for the 22–40 day samples with P values of 0.17 and 0.26 and respectively. Among the PCR assays, NPCR-M showed the highest detection rate for SFTSV in patient samples collected throughout the entire study period, with a minimum detection rate of 70%. The time kinetic study of various PCR assays on SFTS patient samples after symptom onset is presented in Fig. 2.

**Table 1:** Sensitivity and specificity of reverse transcription (RT) polymerase chain reactions (PCR) targeting the M segment of the SFTS virus (single-round PCR-M), RT nested PCR targeting the M-segment of the SFTS virus (NPCR-M), RT-nested PCR targeting the S-segment of the SFTS virus (NPCR-S), and real-time RT PCR targeting the S-segment of the SFTS virus (QPCR-S) conducted in this study for their clinical evaluation of 36 samples from severe fever with thrombocytopenia (SFTS) patient and 85 samples from non-SFTS infectious disease cases.

PCR assay	Single round PCR(M)		N-PCR(M)		N-PCR(S)		q-PCR	
	Case	Control	Case	Control	Case	Control	Case	Control
PCR positive	23	0	36	0	34	0	34	0
PCR negative	13	85	0	85	2	85	2	85
Total	36	85	36	85	36	85	36	85
Sensitivity, % (95% CI)	63.9 (46.2–79.2)		100 (90.3–100)		94.4 (81.3–99.3)		94.4 (81.3–99.3)	
Specificity, % (95% CI)	100 (95.7–100)		100 (95.8–100)		100 (95.8–100		100 (95.8–100)	
PPV, % (95% CI)	100		100		100		100	
NPV, % (95% CI)	86.7 (80.9–91.0)		100		97.7 (91.7–99.4)		97.7 (91.7–99.4)	
AUC (*p* < 0.001)	0.819 (0.739–0.883)		1.000 (0.970–1.000)		0.972 (0.925–0.994)		0.972 (0.925–0.994)	

*CI* confidence interval, *PPV* positive predictive value, *NPV* negative predictive value, *AUC* area under curve.

To compare the sensitivity difference between NPCR-M and real-time QPCR-S, especially in the convalescent stage, samples were tested in duplicate and analyzed as shown in Table 2. The comparison results suggest that the high positivity rate of NPCR-M did not result from contamination. First, a large number of samples presenting high Ct values (39 < Ct < 42) regarded as negative by QPCR-S showed positive results for our designed NPCR-M. Second, the samples regarded as undetermined by QPCR-S were also clearly negative by NPCR-M.

**Table 2:** Comparison of QPCR-S (real-time reverse transcription-RT polymerase chain reaction-PCR targeting the S-segment of the SFTS virus) negative samples (Ct > 42) with NPCR-M (RT nested PCR targeting the M-segment of the SFTS virus) results for blood samples tested from severe fever with thrombocytopenia syndrome (SFTS) patients in the study.

Cycle threshold (Ct)	Day after symptom onset	QPCR-S		N-PCR-M	
		No. of negative samples	(Ct)	Result	No. of positive samples
39 < Ct < 42	–	18	–	–	16/18
	3		40.11	Positive	
	5		39.59	Positive	
	7		39.35	Positive	
	8		40.02	Positive	
	8		41.5	Negative	
	10		41.1	Positive	
	10		39.71	Positive	
	10		39.5	Positive	
	11		39.28	Positive	
	12		40.76	Positive	
	13		39.68	Positive	
	14		39.53	Positive	
	18		39.63	Positive	
	21		40.38	Positive	
	23		39.34	Negative	
	23		40.71	Positive	
	25		41.98	Positive	
	39		40.62	Positive	
42 < Ct	–	5	–	–	1/5
	7		42.07	Negative	
	9		43.15	Negative	
	11		42.03	Negative	
	15		43.96	Positive	
	26		42.99	Negative	
Undetermined	–	12	–	–	0/12

## Discussion

Early and accurate diagnosis of SFTSV infection is crucial for both the survival of patients and prevention of its transmission in the community^9^. Although different PCR methods have been used for the detection of SFTSV by various researchers, data on their comparative potential use for clinical samples are lacking. Our study is the first to design RT-nested PCR targeting the M-segment of the SFTSV and comparing the diagnostic accuracy of different PCRs on clinical specimens collected up to 40 days after disease symptom onset. Through analysis of 38 clinical cases using our developed assay in parallel with the other RT-PCR assays, we demonstrated that our new method has high specificity and higher efficacy than that of the other RT-PCRs, while possessing the potential for increased sensitivity for early detection of the SFTSV. Among the conducted PCR assays, the single-round PCR-M demonstrated a low detection rate for SFTSV in samples collected in the acute and convalescent phases. In contrast, our designed second round of amplification (NPCR-M) substantially enhanced the sensitivity of the assay, as its detection rate remained high throughout the entire study period and it was able to detect positivity in a serologically confirmed patient sample that was deemed negative by all three other assays. QPCR-S had a high detection rate between 1–7 days of symptom onset but then, considerably declined between 8–14 days. NPCR-S exhibited a better detection rate when compared to QPCR-S even after 14 days. Both NPCR (M and S) were capable of detecting SFTSV in some patients even up to 40 days; however, NPCR-M showed an enhanced detection rate throughout the study period when compared to NPCR-S. In addition, NPCR-M conducted for 85 negative control samples of other patients with confirmed viral hemorrhagic fever and various infectious diseases (that need to be differentiated from SFTS) did not show false positive results for any of the samples, indicating the high specificity of this assay.

We further compared the differences in sensitivity between NPCR-M and real-time QPCR-S. The results showed that 16 out of 18 samples presenting values above the cutoff (39 < Ct < 42) showed positive results by NPCR-M, suggesting its high sensitivity. In addition, 12 patient samples undetermined by QPCR-S were also shown to be negative by NPCR-M. This comparison was performed to ascertain the high sensitivity of NPCR-M rather than positive result escalation due to handling contamination. A study conducted on 70 laboratory-confirmed SFTS patients stated that SFTSV viral RNA could be detected in a patient’s serum sample collected even after 20 days after disease onset; however, the optimal sample collection time was within 2 weeks of symptom onset^7^. Our developed NPCR-M showed an enhanced detection rate of 70–80% even between 21–40 days of disease onset. In the SFTS convalescent phase, serological diagnosis provides better sensitivity but in the acute phase, the sensitivity is quite low^7^. We suggest that this developed PCR is suitable for SFTSV infection detection in blood samples collected in the acute and early convalescent phases of the disease, especially where serological tests cannot be performed.

A few samples from different patients presented discordant results. They were tested in duplicate to confirm the differences in the various PCR assay results and then sequenced. We found that the only negative sample by NPCR-M was collected on day 4 of symptom onset and showed negative results for all the PCR assays conducted. However, the follow-up sample for the patient was positive for multiple PCRs and seroconversion was also recorded. This may be due to the patient presenting with SFTS viremia below the detectable limit in the acute phase. Another patient’s initial sample collected on day 7 was only positive by both nested PCRs (NPCR-M and NPCR-S), while QPCR-S and PCR-M results were negative, and follow-up samples were not available for further analysis. In the same manner, the NPCR-S and single-round PCR-M failed to detect one of the patient’s initial (day 5) and follow-up samples collected (day 8), although it was positive for NPCR-M, QPCR-S, and virus was successfully isolated also. In our study, an initial serum sample collected on day 8 yielded a positive result for all the PCR assays except PCR-M; however, the follow-up whole blood sample collected on the next day (day 9) rendered negative results. The results of this study suggest that nested PCR with primers targeting the M-segment of the SFTSV is highly specific and sensitive for the detection of the SFTSV, and it may be useful for the laboratory diagnosis of suspected SFTS. Clinicians can detect SFTSV through nested PCR (as an alternative to other PCRs) if it is suspected that the patient is being seen at the late convalescent phase of the disease.

This study had some limitations. The number of samples collected during each period varied; thus, only a small number of samples were available for the period 22–40 days (n = 10) as compared to the early period. A few of the patients’ follow-up samples were not available for further analysis, as only initial samples were analyzed. Large-scale studies should be conducted in the future to determine the longest period in which SFTSV can be detected via PCR techniques. The risk of contamination cannot be entirely eliminated while conducting nested PCR assays; however, to minimize the carry-over contamination, laboratory protocols were strictly followed.

## Conclusion

Our designed RT-nested PCR targeting the M-segment (NPCR-M) is a highly sensitive and highly specific technique for the diagnosis of the SFTSV in blood samples collected across the acute and early convalescent phases of SFTS.

## Materials and techniques

### Case definition, sample collection, and preparation

We conducted a multicenter prospective study over a period of 8 months (April–November 2018). EDTA whole blood (WB) and serum specimens were collected from 259 febrile patients with suspected SFTS. All patients were aged 18 years or older, visiting Chosun University Hospital or one of 10 other centers. Suspected cases were defined as patients who presented with acute onset of fever (38 °C) or febrile sensation and/or participated in an outdoor activity within the previous month. Laboratory-confirmed cases were defined as suspected cases who fulfilled one or combination of the following criteria: (i) detection of SFTSV RNA by at least two different referenced PCRs, (ii) four-fold increase in IFA titer of IgG-specific antibodies to SFTSV was seen in the paired serum sample, and (iii) SFTSV isolation. Follow-up blood samples were collected up to 40 days (if available). Samples collected at the hospital visit after disease symptom onset within 14 days were considered acute phase samples, and 15–40 day follow-up samples were considered early convalescent phase samples. In addition, 85 EDTA samples, including five blood samples obtained from healthy volunteers and 80 samples from non-SFTS patients with confirmed diseases of other viral or bacterial origin (HFRS = 16, dengue fever = 6, influenza = 7, hepatitis = 3, acquired immune deficiency syndrome [AIDS] = 2, mumps = 4, anaplasmosis = 10, malaria = 2, typhoid fever = 2, syphilis = 4, bacteremia = 12, and others = 12), served as negative controls for testing the specificity of the PCR assays.

Total RNA was extracted from 300µL of whole blood or 150 µL of serum sample using the Viral Gene SpinTM Viral RNA Extraction Kit (iNTRON Biotechnology, Seongnam Korea) according to the manufacturer’s protocol, and cDNA was prepared using the SuperScript VILO MasterMix (Invitrogen, California USA). cDNA was prepared in a total volume of 20 µL by mixing 4 µL VILO MasterMix, 8 µL RNA, and 8 µL distilled water under the following conditions: 25 °C for 10 min, followed by 42 °C for 60 min and 85 °C for 5 min, in a Veriti 96 Well Thermal Cycler (Applied Biosystems, Foster, CA, USA).

### Statement of ethics and compliance

This study was approved by the Ethics in Human Research Committee of Chosun University Hospital (IRB No. 2017-10-012). Written informed consent was obtained from all participants. All methods were approved and carried out in accordance with relevant guidelines and regulations by the institutional review board (IRB) of Chosun University.

### Primers, probes, and PCR

Primers for the M-segment of the SFTSV were designed based on a conserved region of the SFTSV sequence obtained from previous studies and National Center for Biotechnology Information (NCBI). The sequence specificities of the primers were checked by searching the sequences in the GenBank database using the BLAST program. No homology with the sequences of other viral or bacterial organisms was detected. RT-PCR targeting the M-segment (PCR-M) of the SFTSV was performed using the specific primers SFTS-F/SFTS-R as described previously with a small modification^12^. Additionally, RT-N-PCR targeting the M-segment (NPCR-M) of the SFTSV was performed using the first-round primer pair SFTS-M 1st-F and SFTS-M 1st-R for RT-PCR targeting the M-segment, as described above. RT-N-PCR targeting the S-segment (NPCR-S) of the SFTSV was performed using the primers SFTS-S-NP-2F and SFTS-S-NP-2R, and SFTS-S-N2F and SFTS-S-N2R^13^. Quantitative PCR targeting the SFTSV S-segment (QPCR-S) was performed using the primer pair SFTS-SQ-F and SFTS-SQ-R and the SFTS-SQ-P probe^14^. The limit of detection (LOD) was calculated as 96.3 copies nominal with a 95% confidence interval, and cutoff Ct values were taken as < 39. The primers, probes, and conditions used in this study are summarized in Table 3. All oligonucleotide primers and probes were obtained from a commercial source (Bioneer Inc., Daejeon, Korea). The PCR-amplified products were electrophoresed on a 1.5% agarose gel, stained with ethidium bromide, visualized under UV illumination, and photographed.

**Table 3:** Oligonucleotide primers, probes, product base pair (bp) size and conditions used for polymerase chain reactions (PCR) conducted in this study for the detection of severe fever with thrombocytopenia syndrome (SFTS).

PCR assay	Primers and probe name (sequence)	PCR conditions (35 cycles)			Product size (bp)	References
		Denaturing (°C/sec)	Annealing (°C/sec)	Extension (°C/sec)		
SFTS RT-N-PCR (M segment)	SFTS-M 1st-F	95/20	58/40	72/30	510	This study
	(5′-TCATCCTGACYTATTYTGCAATWG-3′)					
(Eexternal primer)	SFTS-M 1st-R					
	(5′- TAAGTYACACTCACACCCTTGAA-3′)					
SFTS RT-PCR	SFTS-F	95/20	58/40	72/30	560	Yun et al.^12^
	(5′-GATGAGATGGTCCATGCTGATTCT-3′)					
(Internal primer)	SFTS-R					
	(5′-CTCATGGGGTGGAATGTCCTCAC-3′)					
SFTS RT-N-PCR (S segment)	SFTS-S-NP-2F	94/20	53/20	72/30	461	Hwang et al.^13^
	(5′-CATCATTGTCTTTGCCCTGA-3′)					
(External primer)	SFTS-S-NP-2R					
	(5′-AGAAGACAGAGTTCACAGCA-3′)					
SFTS RT-PCR	SFTS-S-N2F	94/20	57/20	72/30	346	Hwang et al.^13^
	(5′-AAYAAGATCGTCAAGGCATCA-3′)					
(Internal primer)	SFTS-S-N2R (5′-TAGTCTTGGTGAAGGCATCTT-3′)					
SFTS-real time (S segment)	SFTS-SQ-F	95/05	95/10	51/10	–	Heo, et al.^14^
	(5′-ACCTCTTTGACCCTGAGTTWGACA-3′)					
	SFTS-SQ-R					
	(5′-CTGAAGGAGACAGGTGGAGATGA-3′)					
	SFTS-SQ-P					
	(FAM-5′-TGCCTTGACGATCTTA-NFQ-MGB -3′)					

*bp* base pair, *°C/sec* degrees celsius per second (time).

All PCR assays were performed using the high quality standards required for nucleic acid amplification techniques, including physical separation of the sample preparation area from the PCR mixture preparation and post-amplification areas. All PCR mixtures were prepared under a laminar flow work cabinet irradiated with UV light. Only plugged pipette tips were used to prevent contamination by aerosols, and working surfaces were regularly cleaned using 70% alcohol. During each PCR run, a positive control containing diluted SFTSV cDNA and a negative PCR-grade distilled water control were included. Samples presenting discrepant results in the PCR assays were reanalyzed in duplicate, beginning with re-extraction of RNA.

### PCR diagnostic accuracy assay and statistical analysis

Sensitivity and specificity were calculated using MedCalc program (MedCalc Software Ltd. Ostend, Belgium). The ROC curve was analyzed to determine the diagnostic performance of each PCR conducted, following the method of DeLong et al. (1988) for the calculation of the standard error of the AUC, and an exact binomial method calculating 95% confidence interval for the AUC. The significance level was set at *P* < 0.05. Thirty-six initial samples from the patients collected at the time of hospital admission and 85 confirmed non-SFTS infectious disease samples, as described above, as negative controls were analyzed. Two out of 38 confirmed SFTS patients’ initial samples were excluded for this assay due to their noncompliance with the selection criterion. These two patients had positive PCR results, but they were neither IFA positive nor was virus isolated. To determine and compare the SFTSV detection rate in blood samples (including all 122 initial and follow-up samples) by each PCR assay in relation to days after symptom onset was analyzed using the chi-squared test and statistical significance was set at *p* < 0.05.

### Nucleotide sequencing

Amplified PCR products were purified using QIA quick PCR purification kits (Qiagen, Germany), and direct sequencing was conducted at Macrogen Inc. (Seoul, Republic of Korea) to confirm that the amplified product contained SFTSV sequence.

## Supplementary Information

Supplementary Information.
