# Supplementary material for: Comparison of RT-PCR, RT-nested PCRs, and real-time PCR for diagnosis of severe fever with thrombocytopenia syndrome: a prospective study
Source: Sci Rep. 2021 Aug 18;11:16764. doi: 10.1038/s41598-021-96066-4 (PMC8373928; doi:10.1038/s41598-021-96066-4)
Supplement: Supplementary file 1 — Supplementary Information. [file 41598_2021_96066_MOESM1_ESM.pdf]

Comparison of RT-PCR, RT-Nested PCRs, and Real-time PCR for Diagnosis of Severe Fever with Thrombocytopenia Syndrome: a Prospective Study

Sehrish Jalal[1], Seong Yeon Hwang[2], Choon-Mee kim[3], Dong-Min Kim[2], Na Ra Yun[2]

Jun-Won Seo, [2] Da Young Kim, [2] Sook In Jung,[4] Uh Jin Kim, [4] Seong Eun Kim, [4] Hyun ah Kim,[5] Eu Suk Kim, [6] Jian Hur,[7] Young Keun Kim,[8] Hye Won Jeong,[9] Jung Yeon Heo,[10] Dong Sik Jung,[11] Jieun Kim,[12] Sun Hee Park ,[13] Yee Gyung Kwak,[14] Sujin Lee,[15] and Seungjin Lim,[15] Sun Hee Lee.[16]

Supplementary Figure S1. Flow chart diagram for severe fever with thrombocytopenia syndrome (SFTS) suspect patients and laboratory assays result for 38 confirmed patients with SFTS using initial blood samples.

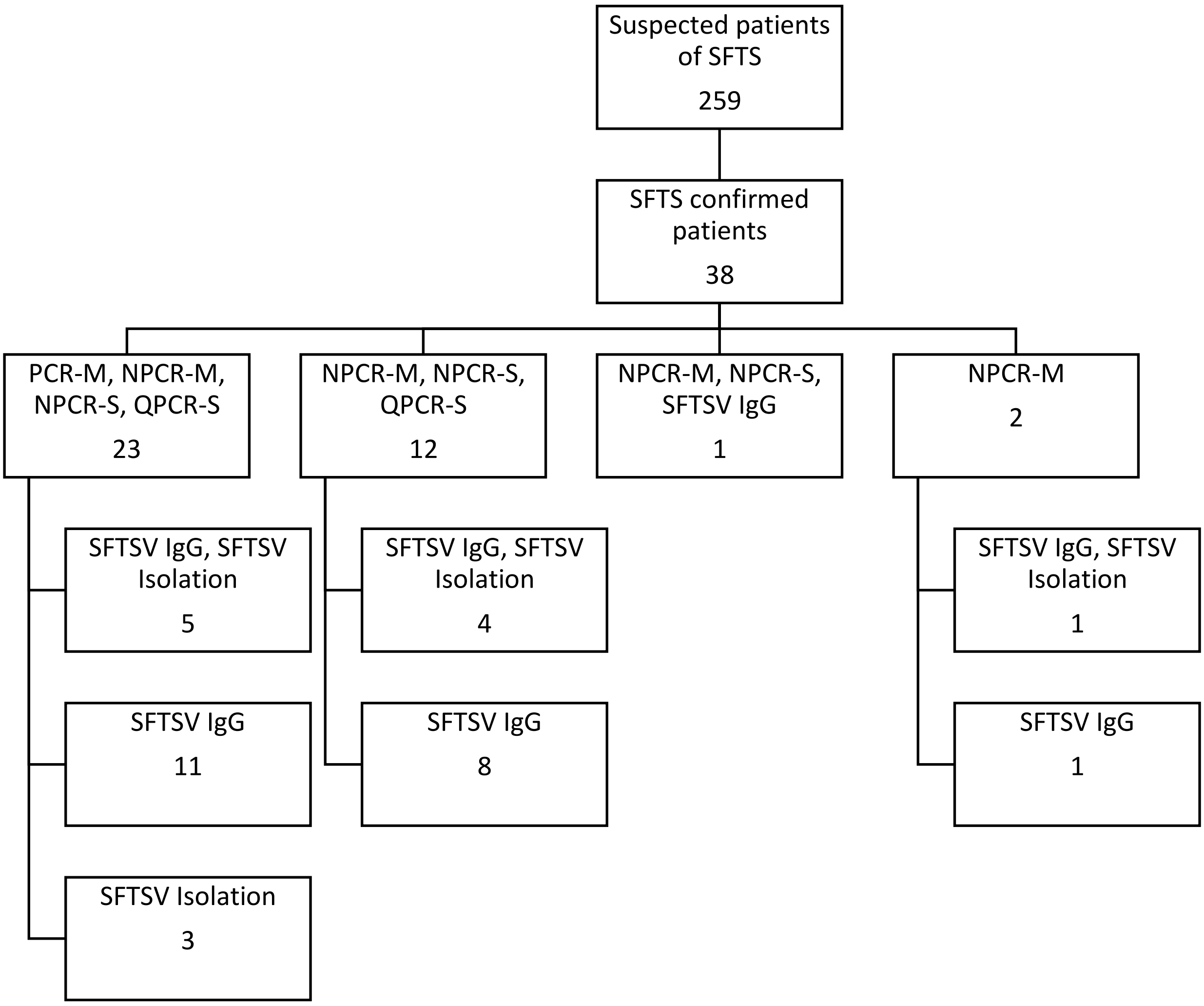

SFTSV: SFTS virus

IgG: Immunoglobulin G

PCR-M = reverse transcription (RT) polymerase chain reaction (PCR) targeting the M segment of the SFTS virus

NPCR-M = RT-nested PCR targeting the M-segment of the SFTS virus

NPCR-S = RT-nested PCR targeting the S-segment of the SFTS virus

QPCR-S = Real-time RT PCR targeting the S-segment of the SFTS virus

Supplementary Table S1. Clinical presentations and laboratory assays result for 38 confirmed patients with severe fever with thrombocytopenia syndrome (SFTS) using initial blood samples.

Patient no.	Clinical presentation			Laboratory assays					
	Fever (38°C)	Outdoor activity	Febrile sensation	QPCR-S (ct)	PCR-M	NPCR-M	NPCR-S	SFTSV IgG	SFTSV isolation
1	Yes	Yes	Yes	31.9	Positive	Positive	Positive	Positive	No
2	Yes	Yes	Yes	35.52	Negative	Positive	Negative	Positive	Yes
3	Yes	Yes	Yes	36.98	Negative	Positive	Positive	Positive	No
4	Yes	Yes	Yes	28.68	Positive	Positive	Positive	Negative	No
5	Yes	Yes	Yes	27.45	Positive	Positive	Positive	Positive	No
6	Yes	Yes	Yes	39.35	Negative	Positive	Positive	Positive	No
7	Yes	Yes	Yes	33.32	Positive	Positive	Positive	Positive	No
8	Yes	Yes	Yes	33.91	Negative	Positive	Positive	Positive	No
9	Yes	Yes	Yes	33.3	Positive	Positive	Positive	Positive	No
10	Yes	Yes	Yes	34.19	Positive	Positive	Positive	Positive	Yes
11	Yes	Yes	Yes	30.32	Negative	Positive	Positive	Positive	No
12	Yes	Yes	Yes	38.36	Negative	Positive	Positive	Positive	Yes
13	NA	NA	Yes	34.19	Positive	Positive	Positive	NA	No
14	Yes	Yes	Yes	29.38	Positive	Positive	Positive	Positive	No
15	Yes	Yes	Yes	34.51	Positive	Positive	Positive	Positive	No
16	Yes	Yes	Yes	37.74	Negative	Positive	Positive	Positive	No
17	Yes	Yes	Yes	24.48	Positive	Positive	Positive	Positive	Yes
18	Yes	Yes	Yes	33.09	Positive	Positive	Positive	Positive	Yes
19	Yes	Yes	Yes	37.42	Negative	Positive	Positive	Positive	No
20	Yes	Yes	No	35.33	Negative	Positive	Positive	Positive	No
21	Yes	Yes	No	28.65	Positive	Positive	Positive	Positive	No
22	Yes	Yes	Yes	30.78	Positive	Positive	Positive	Negative	No
23	Yes	Yes	Yes	35.09	Negative	Positive	Positive	Positive	Yes
24	Yes	Yes	Yes	33.63	Negative	Positive	Positive	Positive	Yes
25	Yes	Yes	No	29.3	Positive	Positive	Positive	Negative	Yes
26	Yes	Yes	Yes	38.91	Negative	Positive	Positive	Positive	No
27	No	Yes	Yes	32.3	Negative	Positive	Positive	Positive	Yes
28	Yes	Yes	Yes	26.4	Positive	Positive	Positive	Negative	No
29	Yes	Yes	Yes	29.35	Positive	Positive	Positive	Positive	Yes
30	Yes	Yes	Yes	26.51	Positive	Positive	Positive	Negative	Yes
31	Yes	Yes	Yes	22.54	Positive	Positive	Positive	Negative	Yes
32	Yes	Yes	Yes	37.04	Positive	Positive	Positive	Positive	No
33	Yes	Yes	Yes	33.25	Positive	Positive	Positive	Positive	No

34	Yes	Yes	Yes	32.12	Positive	Positive	Positive	Positive	No
35	Yes	Yes	No	29.43	Positive	Positive	Positive	Positive	Yes
36	Yes	Yes	Yes	32.66	Positive	Positive	Positive	Positive	No
37	Yes	Yes	No	37.34	Negative	Positive	Positive	Positive	No
38	Yes	Yes	Yes	40.11	Negative	Positive	Negative	Positive	No

°C: degrees Celsius

Ct: cycle threshold

SFTSV: SFTS virus

IgG: Immunoglobulin G

PCR-M = reverse transcription (RT) polymerase chain reaction (PCR) targeting the M segment of the SFTS virus

NPCR-M = RT-nested PCR targeting the M-segment of the SFTS virus

NPCR-S = RT-nested PCR targeting the S-segment of the SFTS virus

QPCR-S = Real-time RT PCR targeting the S-segment of the SFTS virus

NA: no data available
